# Apigenin-Mn(II) loaded hyaluronic acid nanoparticles for ulcerative colitis therapy in mice

**DOI:** 10.3389/fchem.2022.969962

**Published:** 2022-07-22

**Authors:** Feng Lv, Yuchen Zhang, Qiao Peng, Xinxin Zhao, Datao Hu, Jinpeng Wen, Kailai Liu, Ruilin Li, Ke Wang, Jinyao Sun

**Affiliations:** ^1^ School of Pharmacy, Health Science Center, Xi’an Jiaotong University, Xi’an, China; ^2^ Health Science Center, Xi’an Jiaotong University, Xi’an, China; ^3^ Internal Medicine, Xi’an Jiaotong University Hospital, Xi’an, China; ^4^ Department of Pharmacy, The First Affiliated Hospital of Xi’an Jiaotong University, Xi’an, China

**Keywords:** ulcerative colitis, API-Mn(II) complex, regulatory effect, DSS, manganese ion

## Abstract

Ulcerative colitis (UC) is a chronic idiopathic inflammatory bowel disease characterized by rapid progression and frequent comorbidities that make its treatment challenging. Nanomaterial-based strategies have been extensively studied to target the GI mucosal immune system in recent years. Herein, we propose a novel apigenin-Mn(II) loaded sodium hyaluronate nanoparticles where apigenin (API) was incorporated in the Mn^2+^ ramework, coated with hyaluronic acid. The apigenin-Mn(II) loaded sodium hyaluronate nanoparticles (API-Mn(II)@HA NPs) exhibited a diameter of 200 nm and were effective against UC. The preparation of the API-Mn(II) complex was relatively simple, and the mechanism underlying its therapeutic effect on UC induced by sodium dextran sulfate (DSS) was studied in detail. We found that API-Mn(II)@HA nanoparticles could effectively repair the intestinal barrier and significantly improve the damaged colon tissue by mediating inflammatory factors. This study provides novel insights on a new kind of active targeted nanoparticle for improving the efficacy of drugs for UC treatment.

## Graphical Abstract

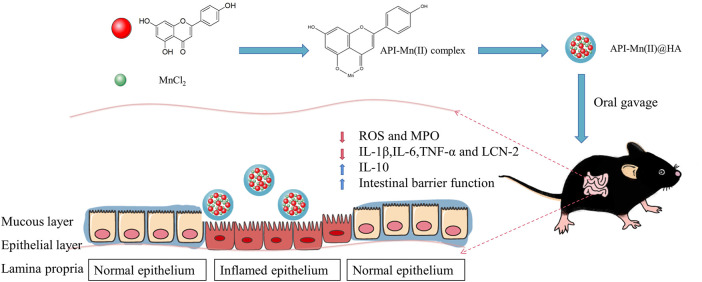

## 1 Introduction

Ulcerative colitis (UC) is a chronic, idiopathic inflammatory disease that affects the colon. It is characterized by a chronic clinical course of recurrent relapse and remission and treatment is often challenging, especially for refractory UC. UC is recognized as one of the modern refractory diseases by the World Health Organization (Gao et al., 2019). The disease area can reach the submucosa of the rectum and involve the rectosigmoid colon and even the whole colon (Molodecky et al., 2012). Its clinical manifestations are abdominal pain, diarrhea, reduced body weight, and hematochezia. An increase in the incidence of UC has been observed in recent years. Given its recurrent and intractable characteristics, it places a major burden on society, becoming a major public health issue. The intricate pathogenesis has been associated to increased mucosal permeability, dysregulation of microorganisms, and inflammatory mediators such as tumor necrosis factor-α (TNF-α), interleukin-6 (IL-6) (Dai et al., 2013).

Given the chronic disease course, patients often require long-term use of drugs such as 5-aminosalicylate sulfasalazine and immunosuppressants. Sulfasalazine (SASP) has become the first-line treatment for ulcerative colitis in China because of its low cost and good efficacy; nonetheless, it has been associated with a high incidence of side effects (Löwenberg and D’Haens, 2013), emphasizing the need for new effective and safe drugs to treat ulcerative colitis. An increasing body of evidence suggests that active ingredients in traditional Chinese medicine, such as polyphenols, alkaloids, quinones, and terpenoids, exert alleviating effects on UC through a multi-target mechanism, with a low incidence of adverse reactions (Shen et al., 2018). Among these, polyphenols have been demonstrated to possess a wide range of biological activity (Zhao et al., 2019). Apigenin (API) is a dihydroflavonoid (4,5,7-trihydroxy flavonoids) extracted from the leaf stem of the Umbelliferaceae plant (Pan et al., 2020). It has been shown that API can inhibit the nuclear factor (Funakoshi-Tago et al., 2011) (NF)-κB activation in the mice induced by carrageenan to reduce the production of inflammatory mediators. Moreover, Ganjare et al. (2011) reported that API could significantly reduce the levels of TNF-α, prostaglandin (PGE_2_), myeloperoxidase (MPO) and malondialdehyde (MDA) in acetic acid-induced lesion model of mice. Yazmin et al. reported that API inhibits the development of sodium dextran sulfate-induced colitis in mice by regulating the typical and atypical inflammatory corpuscle signaling pathways (Márquez-Flores et al., 2016). The above studies suggest that API has huge prospects for application during the treatment of ulcerative colitis. Still, some issues such as poor water solubility, gastrointestinal instability, and poor colonic targeting need to be addressed urgently.

Importantly, hydroxyl and carbonyl groups in the API structure can bind with metal ions to form stable complexes. The complexes can improve API’s water solubility and stability and may enhance its ability to remove free radicals and reduce inflammation. In addition, it has been reported that API-copper (II) complex synthesized in a mixed solvent of ethanol-water has a stronger ability to scavenge free radicals than API (Linglong et al., 2019). These studies indicate that API can enhance stability, water solubility and free radical scavenging ability by binding with metal ions, providing a new approach for applying API to treat inflammatory diseases. Inspired by these findings, we sought to develop a new coordination complex that could significantly refine API’s solubility and free radical scavenging ability. Besides, during intestinal mucosal inflammation, activated epithelial cells, neutrophils and macrophages produce all kinds of inflammatory cytokines and other inflammatory mediators, resulting in impaired function or reduced expression of enzymes that scavenge reactive oxygen species (ROS). When the excessive ROS produced by the intestinal mucosa cannot be removed in time, the balance between the production and elimination of ROS is disrupted. In the meantime, the oxidative damage induced by excessive ROS to DNA, protein and lipid can accelerate the progress of inflammation. Therefore, timely and effective removal of intestinal ROS plays an important role in inhibiting intestinal inflammation. Nanozymes with CAT or SOD mimicking activities can scavenge ROS for neuroprotection, cytoprotection, antiinflammatory, etc, (Yu et al., 2021). There is ample evidence to suggest that a variety of nano-enzymes containing manganese have high ROS scavenging activity (Huang et al., 2016; Yao et al., 2019). Accordingly, Mn^2+^ with strong reducing ability was selected to synthesize a new API-Mn(II) complex with improved reducing and ROS scavenging abilities.

Hyaluronic acid (HA) is a natural non-sulfated polymer mucopolysaccharide that targets immune cells and promotes body repair. In recent years, HA and its derivatives have been widely used as targeting factors in tumor-targeted therapy and body wound repair by specifically binding to CD44 and RHAMM receptors on the cell surface (Collnot et al., 2012). Current evidence suggests that CD44 was overexpressed in colonic epithelial cells and macrophages in ulcerative colitis. It has been shown that macrophages preferentially absorb nanoparticles and can deliver active drugs to the colon inflammatory tissue, given the colonic epithelium’s high permeability and retention effect. Overall, nanoparticles have better targeting ability of the colon, which can effectively reduce adverse reactions and improve bioavailability. Furthermore, scale-up and repeatable production is more onerous if nanoparticle formulation processes contain multistep or complex techniques (Zheng et al., 2021). Therefore, we tried to load the nanoparticles of the APA-Mn (II) complex into HA by a simple method, because HA is an excellent vehicle for improving API targeting capabilities (Lamprecht et al., 2005). In a nutshell, this study sought to improve API’s water solubility and stability by combining it with Mn(II) and improve its targeting effect and bioavailability by HA encapsulation. The mechanisms underlying the therapeutic effect of API-Mn(II)@HA on DSS-induced UC were evaluated in animal experiments. Importantly, our findings provide a theoretical and experimental basis for applying API in UC treatment.

## 2 Experimental section

### 2.1 Materials

Apigenin, manganese chloride, absolute ethyl alcohol and sodium hydroxide were purchased from Xi’en si Co. Ltd, and used without further purification. Hyaluronic acid (HA, molecular weight 90,000–10,000Da, 99%) was purchased from Shandong Furuida Co. Ltd. Dextran sulfate sodium salt (DSS, molecular weight 36,000–50,000Da, 99%) was purchased from MP Biomedicals, LLC and 5-ASA from Hefei Bomei Biological Technology Co. Ltd. ELISA kit was purchased from US Thermo Fisher Scientific. Ultrapure water was prepared using an FDY-1002-UV-P purification system.

60 healthy male C57BL/6 (22 ± 2 g) mice were purchased from the Laboratory Animal Center of Xi’an Jiaotong University. The animals were housed in an animal facility of IVC class (License no. SCXK (Shaanxi) 2015–002) at a controlled temperature of 20–22°C, relative humidity of 50–60% and 12-h day/night cycles. Food and water were provided *ad libitum*. This study was approved by the Ethics Committee of Xi’an Jiaotong University and conducted in accordance with the Guidelines for the Care and Use of Laboratory Animals issued by the Ethics Committee of Xi’an Jiaotong University, China (License No.: XJTU 2019–003).

### 2.2 Methods

#### 2.2.1 Preparation and characterization of API-Mn(II) complex

0.25 mmol API was dissolved into 30 ml ethanol and stirred at a rate of 1000 r/min for 20 min at 60°C. 0.25 mmol manganese chloride was added to 5 ml ethanol and shaken until completely dissolved, and drops of the mixture were added to the API ethanol solution. NaOH solution (1 mol/L) was used to adjust the pH of the solution to 8. Then brown-yellow precipitates were observed in the solution. After stirring at 60°C for 4 h, the reaction was stopped, and the product was filtered and dried to obtain a coarse product. These coarse products were dissolved in water, filtered to remove the undissolved ingredients, and lyophilized to obtain a brown-yellow solid product (Li et al., 2016).

To better determine the mix ratio between API and Mn(II), the molar ratio method in spectrophotometry was used to determine the mix proportion. 0.5 mmol/L API ethanol solution and manganese chloride ethanol solution were prepared. The volume of API ethanol solution in each sample was 1.2 ml, and the volume of manganese chloride ethanol solution was successively changed from 0 to 2.0 ml. There were ten groups, and the final constant volume of each sample solution was 5.0 ml. The absorbance A of each sample at the maximum absorption wavelength of 380 nm was detected to obtain an absorbance curve A associated with C_M_/C_L_ (Pan et al., 2014). With an increase in C_L_, the concentration of the complex and the absorbance A were increased. When a turning point was observed on the curve, the C_M_/C_L_ value corresponding to the turning point was regarded as the mix proportion of the complex.

#### 2.2.2 Preparation of API-Mn(II)@HA nanoparticles

10.0 mg API-Mn(II) and 60.0 mg HA were accurately weighed and dissolved in 40 ml deionized water. Then the reactants were dispersed evenly using ultrasonication. 1 mol/L NaOH solution was used to adjust the pH value of the mixed solution to 8-9 to obtain a light yellow solution containing a small amount of black precipitate. The reaction was conducted at room temperature (25°C) for 24 h with a magnetic agitator at 600 r/s in a dark environment. The solution was filtered, and the supernatant was centrifuged at 8000 r/s for 5 min to obtain a light yellow solution on the upper layer and a black solid on the bottom layer. The supernatant was again filtered, and a yellow flocculent product was obtained after lyophilization (Zhu et al., 2012). In order to determine the feeding ratio of hyaluronic acid, we adjusted the ratio of the complex to HA to 1:3, 1:6, and 1:9, respectively, and compared the appearance characteristics, yield, drug loading rate, and infrared characteristics of the three products. It has distinct infrared characteristics, uniform appearance, color and texture, and higher yield and drug loading rate.

#### 2.2.3 Characterizations of polymers

The polymer products were characterized by Fourier transform infrared (FT-IR), particle size test and TEM.

The FT-IR spectra of the lyophilized API-Mn(II)@HA nanoparticles were recorded on an FT-IR spectrometer (Nicolet 6700, Thermo Fisher) in the frequency range of 4000–500 cm^−1^ with 32 scans and a resolution of 4 cm^−1^.

#### 2.2.4 Animals experiments

60 mice were divided into 6 groups (10 mice per group), namely a normal control group (Control Group), DSS model control group (Model Group), Apigenin group (API Group), Apigenin-Mn(II) group (API-Mn(II) complex group), Apigenin-Mn(II)@HA group (API-Mn(II)@HA nanoparticles group) and 5-aminosalicylic acid group (5-ASA group). The mice were housed in an animal facility of IVC class [License no. SCXK (Shaanxi) 2015-002] at a controlled temperature of 20–22°C, relative humidity of 50–60% and 12-h day/night cycles. Food and water were provided *ad libitum*. The entire experiment period was conducted for 9 days 2% Dextran Sulfate Sodium (DSS) was added to the drinking water to establish the mice models of acute ulcerative colitis while mice in the control group only received drinking water. 5-aminosalicylic acid (5-ASA), widely used in the clinical treatment of UC and API raw materials, were selected for comparison. The mice’s physiological status and body weight were recorded daily. The fecal status was observed, and the disease activity index (DAI) was scored (Supplementary Table S1). After 9 days, the mice were killed and dissected immediately. The feces were collected, and the colon length of mice in each group was measured. The thymus and spleen of mice in each group were harvested and weighed after the residual blood was sucked by filter paper. Then the thymus index and spleen index were calculated in detail.

#### 2.2.5 Histopathological examination

The colon sections were processed for histopathological examination. After the mice were killed, the colon tissues were harvested and fixed with 4% paraformaldehyde solution. 5μm-thick paraffin-embedded tissue sections were obtained and stained with H&E and Masson reagent kit, respectively. Two different histopathologists evaluated the histopathological colon scores. The inflammation, extent (Sakai et al., 2019), crypt damage and area involved as multiplication factors were included in the histopathological colon score (Supplementary Table S2).

Biosafety is a critical issue for the biological application of nanomaterials. Therefore, the biodistribution of Ti3C2 NSs in major organs of mice was investigated.

#### 2.2.6 MPO analysis

Colon tissue was washed in cold saline and ground to make a homogenate on pre-cooled PBS. A 10% homogenate was made by adding PBS at a volume of 1:9, and the corresponding enzyme-linked immunosorbent assay kit was used to detect MPO enzyme activity.

#### 2.2.7 Oxidative stress indexes analysis

Colon tissue was taken, rinsed with frozen saline, dried with filter paper and weighed. The colon tissue was cut up and homogenized in normal saline (at a volume of 1:9) to prepare 10% homogenate in an ice bath. Then the corresponding enzyme-linked immunosorbent assay kit was used to assess the antioxidant enzyme activities of SOD, MDA, GSH-Px, and CAT, respectively.

#### 2.2.8 Inflammatory cytokines analysis

The colon tissue was homogenized, centrifuged, and the supernatant was collected. The corresponding enzyme-linked immunosorbent assay kit was used to detect the levels of TNF-α, IL-1β, IL-6, IL-10, IFN-γ, IL-17, and TGF-β1, respectively. The procedure was carried out in strict accordance with the manufacturer’s instructions (Márquez-Flores et al., 2016).

#### 2.2.9 Immunofluorescence histochemistry

On day 8 of the experiment, the colon tissue of mice was taken, the homogenate was cleavage, and the protein was extracted. The analysis of ZO-1 and Occludin-1 expression was performed *via* immunofluorescence staining. In short, paraffin-embedded tissue sections (5 μm) were deparaffinized, rehydrated and washed with 1% PBS. The membrane was sealed in 3% BSA at 25°C for 30 min. The primary antibody was diluted with BSA at a specific proportion and incubated overnight at 4°C. HRP-labeled secondary antibodies were added and incubated at room temperature for 50 min. The slides were incubated with TSA-FITC solution (diluted with TBST appropriately) for 10 min in the dark after microwave treatment. Then, the second primary antibody and corresponding secondary antibody were added. Finally, the slides were then counterstained with DAPI at room temperature for 10 min in the dark. Images were acquired by a fluorescence microscope.

#### 2.2.10 Statistical analysis

The statistical analysis was performed using SPSS15.0. The results were expressed as the mean ± SD. One-way ANOVA analysis was conducted to assess the significance of differences in three or more groups using GraphPad Prism 8. Statistical significance was indicated as *p < 0.05, **p < 0.01, ***p < 0.001 and ****p < 0.0001.

## 3 Results and discussion

### 3.1 Synthesis of API-Mn(II)@HA nanoparticles

To improve the solubility of API and explore its potential application in ulcerative colitis treatment, we synthesized a novel API-Mn(II) complex prepared by metal ion complexation and factors that influenced the synthesis process were explored in detail. It is widely acknowledged that increasing the temperature can accelerate the reaction speed, but excessively high temperatures may destroy the structure of API, thus affecting its complexation ability. The yield of API-Mn(II) complex (Y) was taken as the response value, and pH (A), time (B) and temperature (C), three factors that significantly affected the yield, were considered as the investigation factors. The factors and levels of the Box-Behnken test are shown in Supplementary Table S3, and the test results and analysis data are shown in Supplementary Tables S4, S5

**FIGURE 1:**
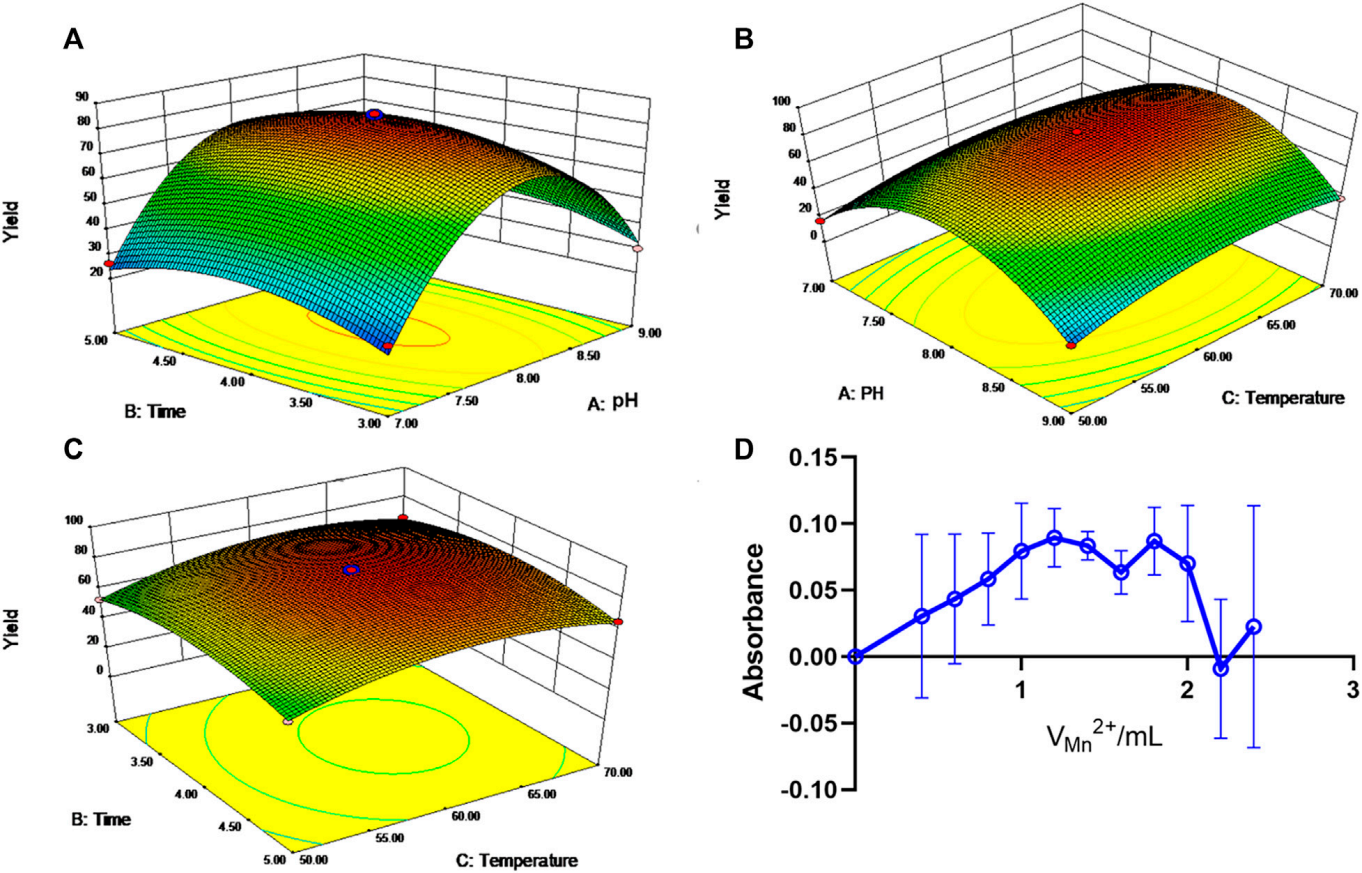
Response surface and contour line of the influence of each factor interaction on yield. **(A)**. Response surface of pH and time to yield. **(B)**. Response surface and contour of pH and temperature effects on yield. **(C)**. Response surface and contour lines for the effect of time and temperature on yield. **(D)**. The curve of the molar ratio method.

The response surface curves and contour lines of the API-Mn(II) complex product under different reaction temperatures, pH and reaction times are shown in Figure 1. A regression equation was obtained by carrying out regression analysis using Design Expert 8.0.5 software. The optimal process parameters were then determined, A reaction time of 4 h at a temperature of 62.2°C and pH 8.07 obtained a yield of 83.13%. For convenience, the optimal process parameters were modified to reaction time 4.00 h, temperature 60°C and pH 8. Under optimal conditions, the yield was 81.68%, consistent with the model’s predicted value (83.13%), substantiating the reliability of our model.

As shown in Figure 1D, at C_M_/C_L_ = 1, the absorbance A reached a peak value, suggesting that manganese ion and API form a 1:1 complex.

The yield of the complex with different mix proportions and the amount of HA are provided in Supplementary Table S6. Colors of the final products with the three different ratios of HA were different. The color of the products of the 1:3 group was not uniform, and black solids were observed, which might be due to an insufficient amount of HA to encapsulate the drug completely. However, the yield of the 1:9 group was the lowest, and the product exhibited a strongly viscous shape, which might be due to excessive HA. Furthermore, the encapsulation and drug loading rates of the complex with different mix proportions of HA are provided in Supplementary Figure S1. Finally, the ratio of 1:6 was chosen as the optimal ratio for the reaction between the API-Mn(II) complex and HA. API-Mn@HA greatly improves the solubility of the API, and UV results show that the water-solubility of API-Mn@HA increases by 41.5 times (Supplementary Figure S4).

### 3.2 Characterizations of polymers

**FIGURE 2:**
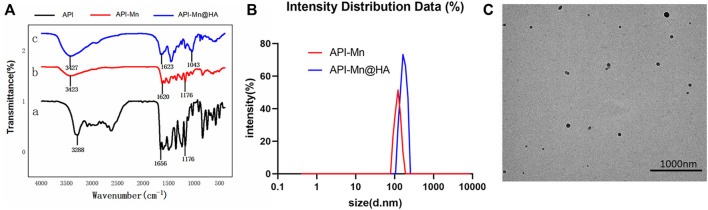
Characterizations of polymers. **(A)**. Infrared spectrogram of API-Mn(II) complex and API-Mn(II)@HA nanoparticles. [a.API, b.API-Mn(II)@HA nanoparticles, c.API-Mn(II) complex] **(B)** Size distribution of API-Mn and API-Mn@HA. **(C)** TEM images of API-Mn@HA.

As shown in Figure 2A, FT-IR analysis showed that the characteristic peak at 3288 cm^−1^ belonging to the stretching vibration of the phenolic hydroxyl group changed to a strong and wide absorption peak, indicating that the formation of the complex between API and Mn(II) was related to hydroxyl groups. A strong absorption peak of the generated API-Mn(II) complex was observed at 3423 cm^−1^, showing the presence of water molecules in the complex. The strong stretching vibration peak of the carbonyl group at the 4-position of API shifted from 1656cm^−1^ to 1620cm^−1^, and the peak was smaller. This finding may be attributed to the deviation of the carbonyl bonding electron from the geometric center of the bond to the side of the oxygen atom after coordination bonding between the oxygen atom on the carbonyl group and Mn(II), finally reducing the density of the carbonyl bonding electron cloud and causing a blue shift of the peaks. Therefore, it can be concluded that carbonyl oxygen at the 4-position is involved in coordinate bonding, forming the C-O-Mn(II) structure. Both API and complex exhibited strong absorption peaks at 1176 cm^−1^, and the vibration frequency of the ether bond was not changed, indicating that the cycloether bond structure was not damaged (Li et al., 2016). As seen in Figure 2B, the particle size of API-Mn was approximately 100 nm. After complexing with HA, the particle size distribution shifted to the right to values slightly greater than 100 nm. The TEM images (Figure 2C) clearly showed that the complex combined with HA to form a sphere.

### 3.3 Therapeutic effects of API-Mn(II)@HA nanoparticles on ulcerative colitis

**FIGURE 3:**
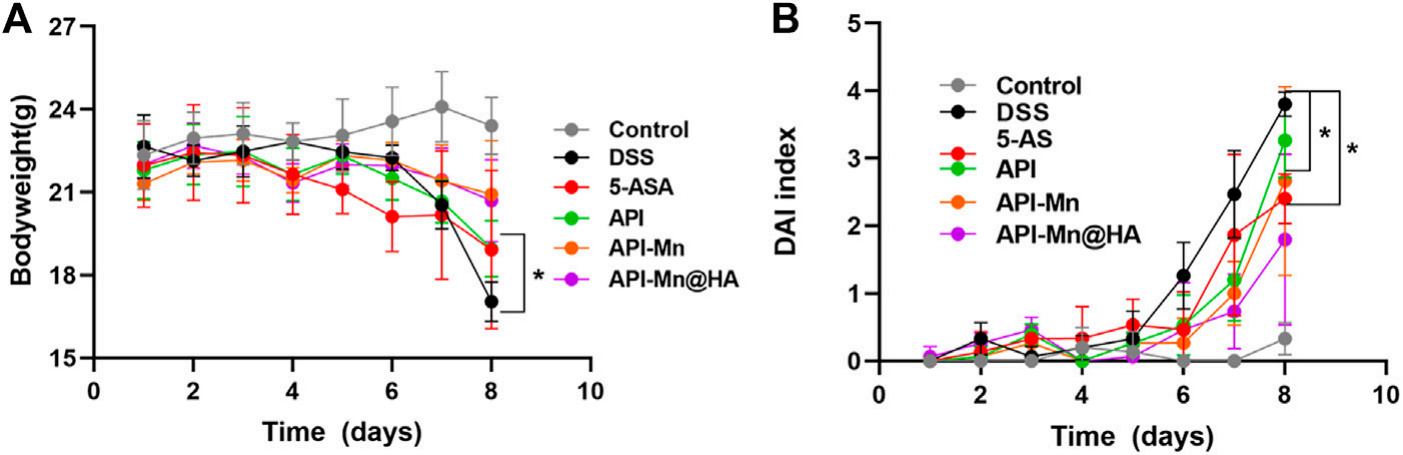
Effects of API manganese complex and API manganese complex coated with HA on physiological state of mice induced by DSS. **(A)** Curve of changes in DAI in mice. **(B)**. Curve of changes in bodyweight in mice. Data are shown as mean ± SD. *n* = 5, *p < 0.05, **p < 0.01, ***p < 0.001 and ****p < 0.0001.

In the present study, 2% DSS solution was used to establish mice models of UC (Supplementary Figure S3). During the experiment, mice in the control group exhibited normal behavior (feeding, grooming, and social interactions) with smooth hair, normal fecal characteristics, a stable increase in body weight, and a clinical DAI index of 0. Mice in the DSS group often assumed a hunched position with erect hair exhibiting sparse growth and exhibited decreased food and water intake. From day 6, all mice presented with hematochezia. The body weight remained stable for the first 4 days and began to decrease from day 5. The DAI index remained stable in the first 4 days, increased slightly on day 5, and then increased substantially for the following 3 days. After DSS was used to induce UC, the colitis-related symptoms of the other four groups of mice were significantly relieved. As shown in Figure 3A, the body weight of mice in the 5-ASA, API-Mn(II) complex and API-Mn(II)@HA groups were increased to a certain extent. API-Mn(II) complex and API-Mn(II)@HA nanoparticles groups yielded similar results but were statistically significantly higher than in 5-ASA and API groups from day 6 (*p* < 0.05).

According to Figure 3B, after DSS was used to induce UC, the DAI scores of the other four groups of mice were lower than the DSS group. Compared with the DSS group, the API-Mn(II)@HA nanoparticles group exhibited the most significant decrease in DAI score, and the API group had the highest DAI score (*p* < 0.05). In conclusion, our API-Mn(II)@HA nanoparticles could improve the symptoms of DSS-induced colitis and exert a therapeutic effect.

### 3.4 Effects of API-Mn(II) complex and API-Mn(II)@HA nanoparticles on colon length, thymus and spleen indexes in DSS-induced colitis

**FIGURE 4:**
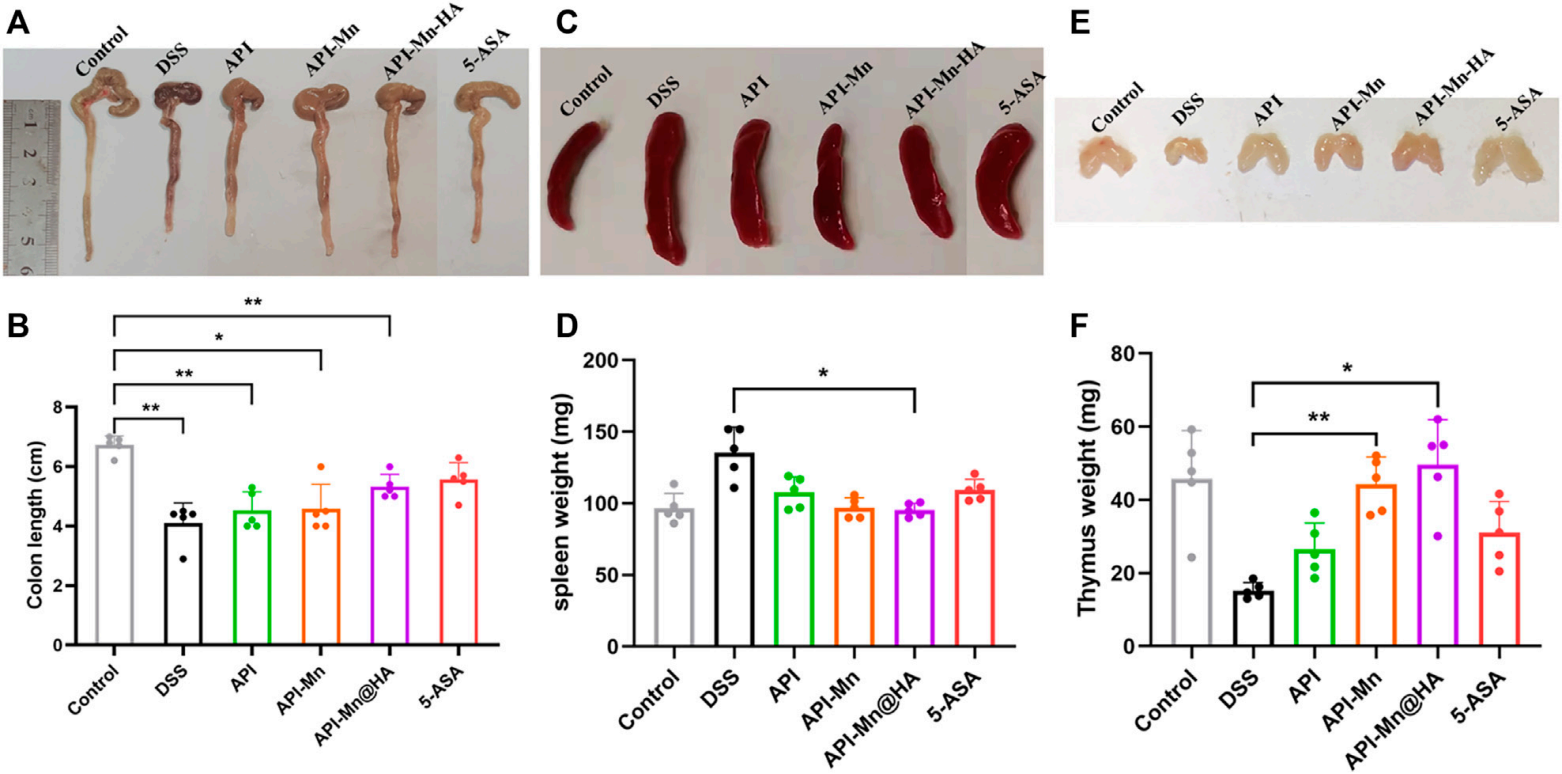
Effects of API manganese complex and API manganese complex coated with HA on colon length and thymus and spleen indexs in DSS induced mice. Healthier mice have longer colons, lighter spleens and heavier thymuses. **(A,B)** Comparison of colon length in all the groups. **(C,D)** Comparison of spleen weight in all the groups. **(E,F)** Comparison of thymus weight in all the groups. Results in 5-ASA group and API-Mn@HA group are much better compared with that of DSS group. Data are shown as mean ± SD. *n* = 5, *p < 0.05, **p < 0.01, ***p < 0.001 and ****p < 0.0001.

It has been established that DSS-induced colitis in mice causes congestion and swelling of the colon, leading to intestinal wall thickening, increased colon weight and shorter colon length. Therefore, the length of the colon can directly reflect the severity of colitis and drug efficacy. As shown in Figures 4A,B, the colon length was significantly shortened after DSS-induced colitis and improved after administration of API-Mn(II)@HA nanoparticles and 5-ASA. No difference was found between API-Mn@HA and 5-ASA. As shown in Figure 4C, DSS induced decreased thymus weight in mice, and increased thymus weight was observed in the API-Mn API-Mn@HA group (*p* < 0.05). As shown in Figure 4D, the API-Mn@HA group exhibited a significant inhibitory effect on spleen inflammation (*p* < 0.05). Overall, our findings suggest that API-Mn(II)@HA nanoparticles exert a protective effect against DSS-induced colon length shortening in UC mice and a therapeutic effect on UC (Huang et al., 2016).

### 3.5 Effects of API-Mn(II) complex and API-Mn(II)@HA nanoparticles on pathological characteristics of colon tissues in DSS-induced colitis

**FIGURE 5:**
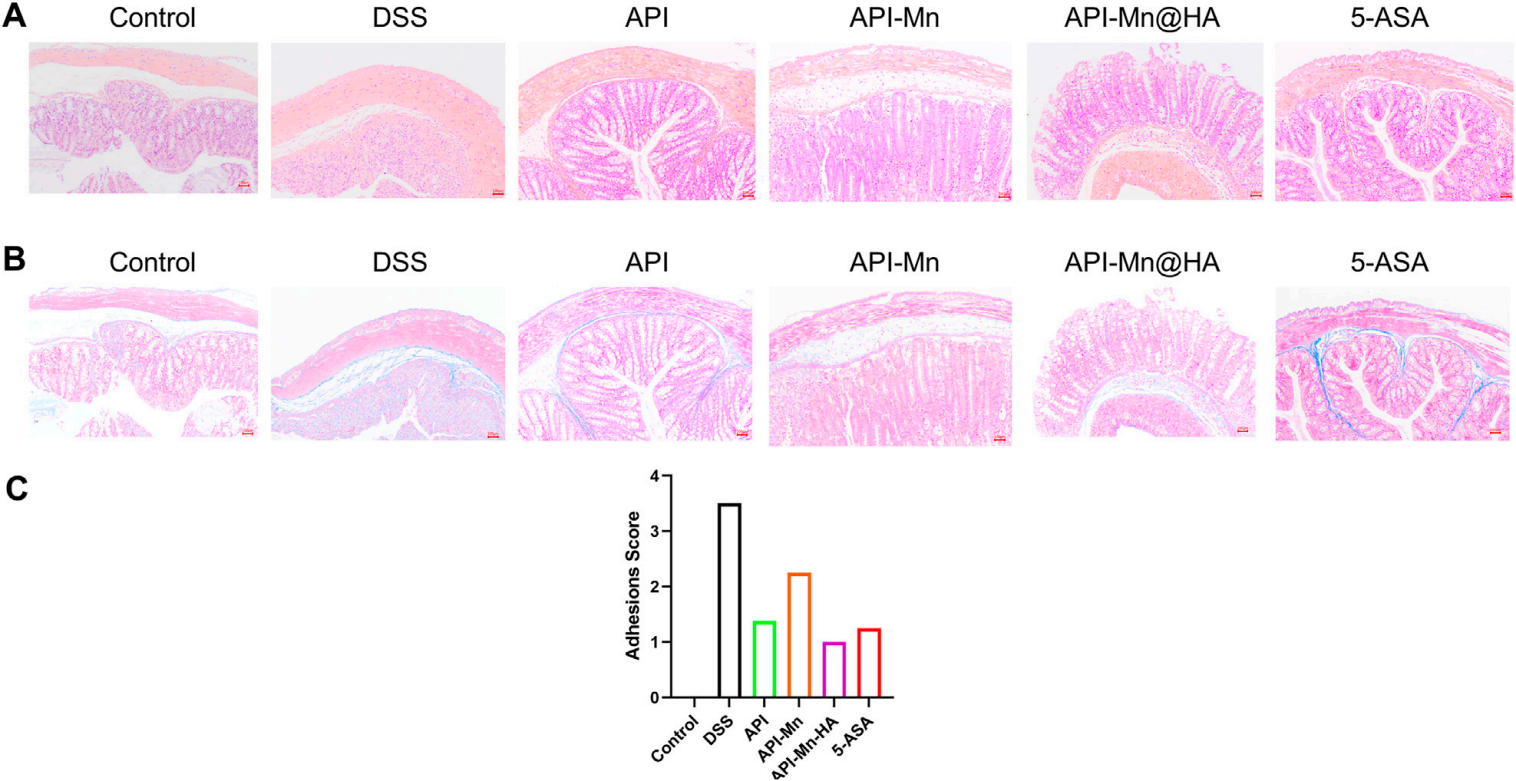
**(A)** Histopathological changes of colon by HE staining. **(B)** Masson staining. **(C)** Adhesions score of inflammation of the colon. (Magnification is 400 times and the scale is 100 nm) Data are shown as mean ± SD. *n* = 5, *p < 0.05, **p < 0.01, ***p < 0.001 and ****p < 0.0001.

H&E and Masson staining were conducted on colon tissue sections in each group. The results were observed under a light microscope and photographed (Figure 5A **)**. In contrast with the control group, colon tissue of mice in the DSS model group exhibited significant mucosal erosion, ulcers, and subcutaneous gland atrophy with no goblet cell visible. The intestinal lumen was barely visible due to extensive cell and tissue disintegration, high inflammatory cell invasion and significant tissue edema. As seen in Figure 5B **,** compared with the DSS group, decreased inflammatory cell invasion was observed in various intestinal acini with normal goblet cell numbers. The lumen and secretions could be clearly observed. The shedding and disordered arrangement of tissue cells caused by intestinal mucosal injury were alleviated. According to Figure 5C, the histological scores of the other four groups of mice treated with DSS were lower than in the DSS group. The 5-ASA and API-Mn(II)@HA nanoparticles groups exhibited the lowest histological scores, while the API group yielded the worst performance. API-Mn(II) complex and API-Mn(II)@HA nanoparticles exerted a protective effect against colitis and could significantly improve the pathological damage caused by ulcerative colitis.

In addition, Hematoxylin and eosin (H&E) staining, Masson staining and histological analysis also showed no obvious tissue damage or adverse reactions in the API-Mn@HA group (Supplementary Figure S2). These data showed that API-Mn@HA had no significant side effects in mice.

### 3.6 Effects of API-Mn(II) complex and API-Mn(II)@HA nanoparticles on MPO activity in DSS-induced colitis

**FIGURE 6:**
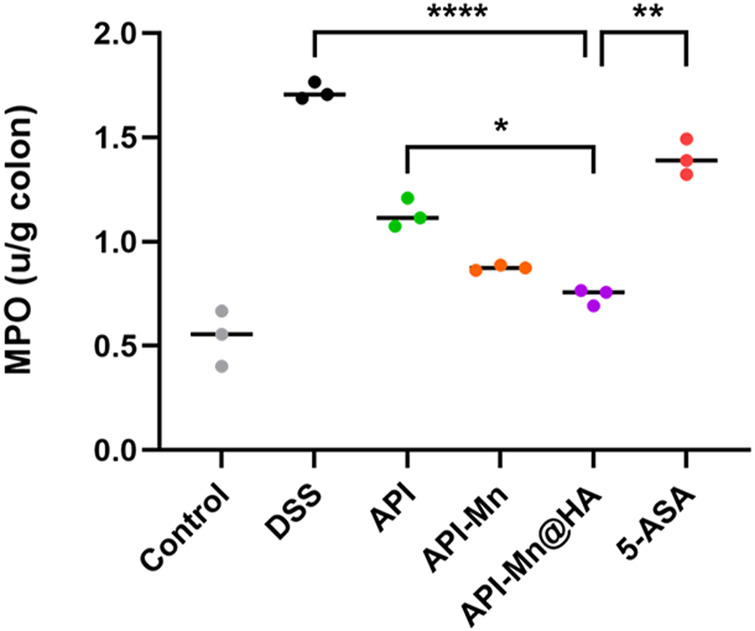
Histogram of the activity of MPO, a marker of the activation and function of neutrophils. Data are shown as mean ± SD. *n* = 5, *p < 0.05, **p < 0.01, ***p < 0.001 and ****p < 0.0001.

It has been established that MPO is a neutrophil activation and function marker. Its level and activity changes reflect the functional and activation status of neutrophilic polynucleated white blood cells (PMN). In the present study, the MPO activity in colon tissue was measured by protein extraction. We found that after DSS stimulation, MPO activity in mice colon tissue increased from 0.542 ± 0.109 IU/g to 1.720 ± 0.034 IU/g, suggesting that neutrophils in colon tissue were significantly activated. As shown in Figure 6, MPO activity of colon tissue in the API, API-Mn(II) complex and API-Mn(II)@HA nanoparticles groups were significantly reduced to 1.133 ± 0.057 IU/g, 0.875 ± 0.010 IU/g and 0.739 ± 0.032 IU/g (*P*_*s*_ < 0.01). Compared with the DSS group, the API-Mn(II)@HA nanoparticles group exhibited significantly lower MPO activity (*p* < 0.0001), similar to the API-Mn(II) complex group, and higher than the 5-ASA group (*p* < 0.05).

### 3.7 Effects of API-Mn(II) complex and API-Mn(II)@HA nanoparticles on oxidative stress indexes in DSS-induced colitis

**FIGURE 7:**
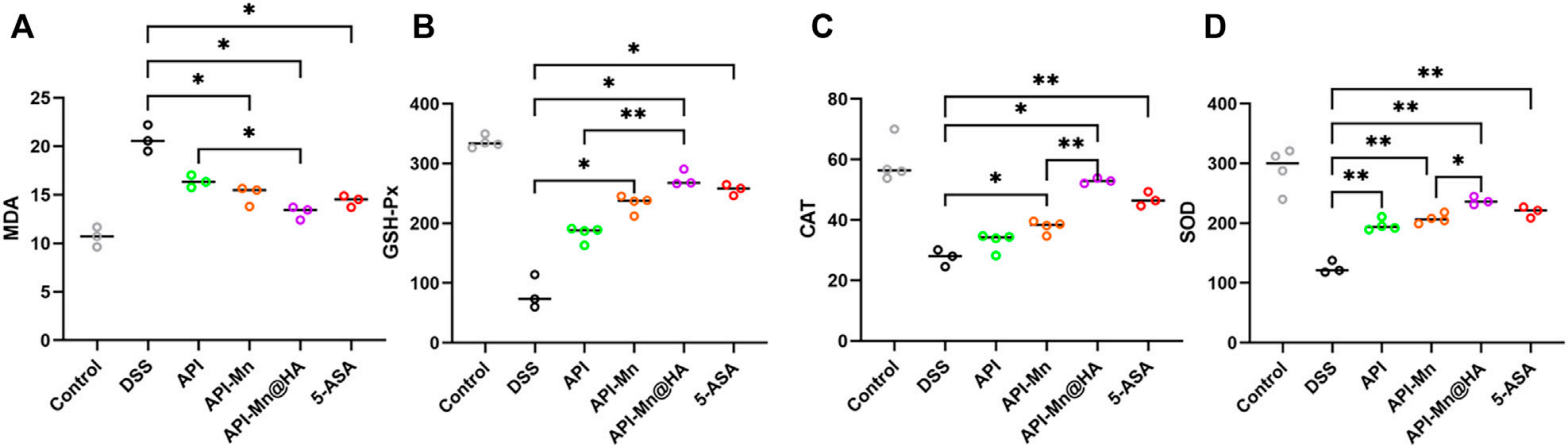
Effects of API-Mn(II) complex and API-Mn(II)@HA nanoparticles on the oxidative stress index in DSS-induced mice. **(A)** The MDA activity of colon tissue of mice in DSS group is much higher than other groups, showing more severe inflammatory response. **(B)** The SOD activity of colon tissue of mice. **(C)** The CAT activity of colon tissue of mice. (D). The GSH-Px activity of colon tissue of mice. Data are shown as mean ± SD. *n* = 5, *p < 0.05, **p < 0.01, ***p < 0.001 and ****p < 0.0001.

Malondialdehyde (MDA) is well-recognized as one of the end products of membrane lipid peroxidation. Its content can be used to evaluate the degree of cell injury. In the present study, the MDA activity of each group was measured using ELISA. As shown in Figure 7A, the MDA content in colon tissue was higher in the DSS group than in the control group (20.77 ± 1.12 nmol/ml vs. 10.94 ± 0.85 nmol/ml), indicating that DSS stimulation could cause significant injury to colon tissue. Compared with the DSS group, the MDA content in colon tissue of the API, API-Mn(II), API-Mn(II)@HA nanoparticles and 5-ASA groups was significantly lower (14.37 ± 0.49 nmol/ml, 16.77 ± 0.80 nmol/ml, 15.50 ± 1.16 nmol/ml, and 12.27 ± 1.86 nmol/ml, respectively) (*P*_*s*_ < 0.05). This finding suggested that all drugs used in the experiment could effectively scavenge free radicals, alleviate intestinal mucosal damage and protect the colonic mucosal barrier.

SOD, GSH-Px, and CAT are important antioxidant enzymes that play an important role in attenuating free radical-induced oxidative stress. Importantly, SOD and GSH-Px can catalyze the reduction of oxygen free radicals and generate hydrogen peroxide, which is further reduced to water molecules by CAT (Balmus et al., 2016). As shown in Figure 7B, GSH-Px activity in colon tissue of mice in the DSS group was significantly reduced compared with the control group (82.61 ± 3.85 U/ml vs. 335.97 ± 8.53 U/ml), indicating that DSS stimulation could significantly impair the colon’s antioxidant capacity. GSH-Px activity in colon tissue of the API-Mn(II) and 5-ASA groups was significantly higher than in the DSS group (*p* < 0.05), and higher in the API-Mn(II)@HA nanoparticles group than in the API group (*p* < 0.05), indicating that API-Mn(II)@HA nanoparticles induced a more significant anti-inflammatory effect than API.

According to Figure 7C, compared with the control group, CAT activity of colon tissue was lower in the DSS group (59.16 ± 6.38 U/ml vs. 27.57 ± 2.29 U/ml), indicating that DSS stimulation could impair the colon’s antioxidant capacity. CAT activity of colon tissue in the API-Mn(II) complex and 5-ASA groups was significantly higher than in the DSS group (*p* < 0.05). Moreover, a significant difference in CAT activity between API-Mn(II)@HA nanoparticles group and API-Mn(II) complex group was found (*p* < 0.05), indicating a better anti-inflammatory effect after drug encapsulation with HA.

As shown in Figure 7D, SOD activity in colon tissue of mice in the DSS group was lower than in the control group (125.61 ± 8.68 U/ml vs. 290.33 ± 31.4 U/ml), indicating that DSS stimulation severely decreased the colon antioxidant capacity, consistent with the findings for GSH-Px and CAT. SOD activity in colon tissue of the API, API-Mn(II) complex, API-Mn(II)@HA nanoparticle and 5-ASA (219.16 ± 7.59U/mL, 196.95 ± 8.33U/mL, 207.57 ± 7.02U/mL, and 237.35 ± 5.65U/mL) groups was significantly higher than in the DSS group (125.61 ± 8.68U/mL) (*P*_*s*_ < 0.05). Moreover, a significant difference in SOD activity was found between the API-Mn(II)@HA nanoparticle group and the API-Mn(II) complex group (*p* < 0.05), indicating a better anti-inflammatory effect after HA encapsulation, as previously demonstrated for CAT activity. In conclusion, compared with the DSS group, SOD, GSH-Px and CAT activity in API-Mn(II) complex and API-Mn(II)@HA nanoparticles groups were increased (*p* < 0.05), suggesting that API-Mn(II) complex and API-Mn(II)@HA nanoparticles could significantly improve the colon’s antioxidant capacity and reduce oxidative damage.

### 3.8 Effects of API-Mn(II) complex and API-Mn(II)@HA nanoparticles on the regulation of inflammatory factors in DSS-induced colitis

**FIGURE 8:**
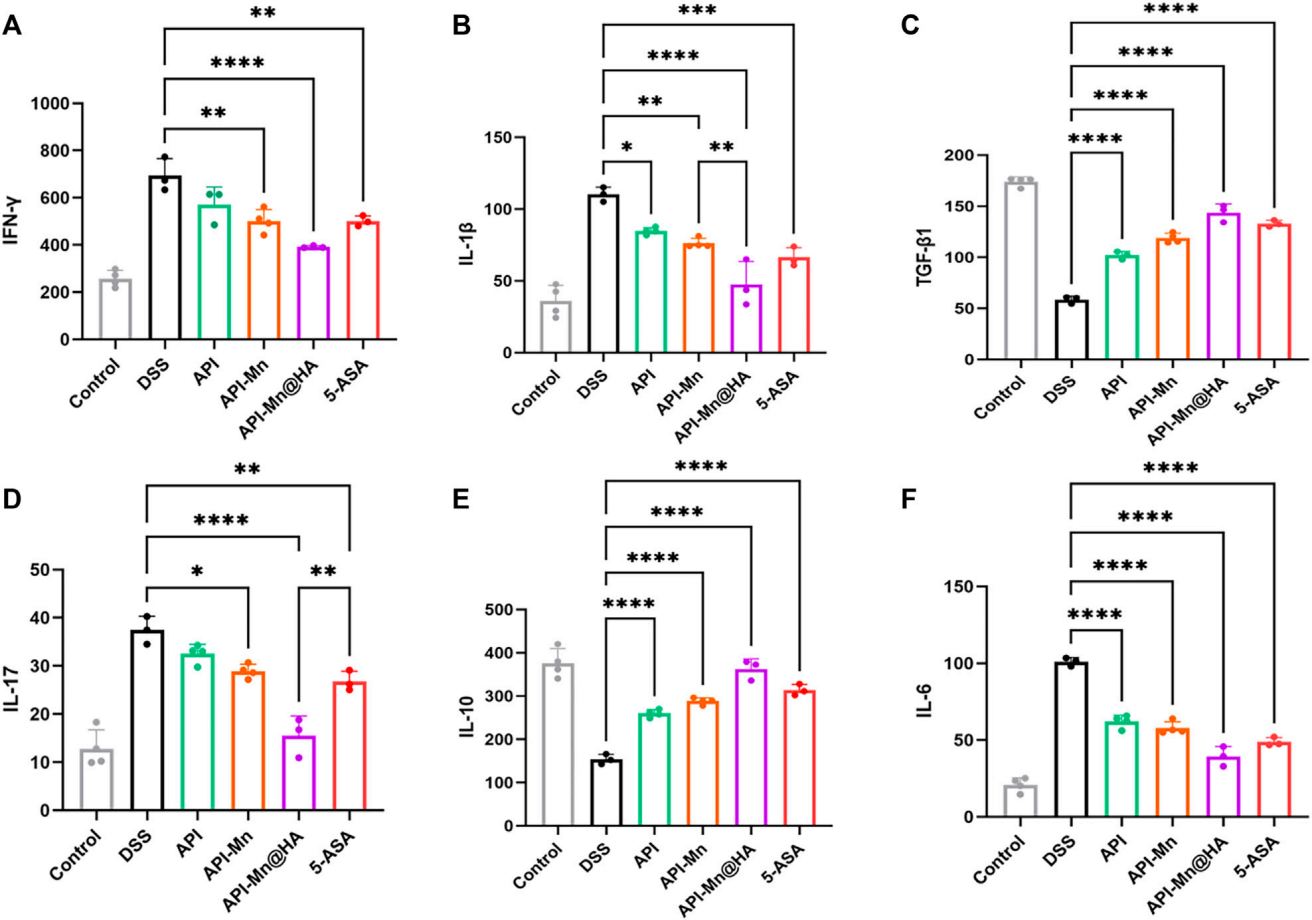
Effects of API-Mn(II) complex and API-Mn(II)@HA nanoparticles on the inflammatory factors in DSS-induced mice. Inflammatory factors INF-γ **(A)** , IL-1β **(B)** , TGF-β1 **(C)** , IL-17 **(D)** , and IL-6 **(F)** were significantly reduced in the experimental group, while the anti-inflammatory factor IL-10 **(E)** was significantly increased. Data are shown as mean ± SD. n = 5, *p < 0.05, **p < 0.01, ***p < 0.001 and ****p < 0.0001.

Current evidence suggests that inflammatory cytokines mediate many immune responses in UC. Nonetheless, the exact pathogenic mechanism of these small peptides remains unclear. It is believed that the balance of proinflammatory and anti-inflammatory cytokines in the intestinal mucosa is critical for maintaining intestinal balance. Excessive production of proinflammatory cytokines such as TNF-α, IL-6, IL-1, IL-12, and IL-23, and insufficient anti-inflammatory cytokines such as IL-4, IL-10, IL-19, and IL-22 lead to a dysregulated intestinal environment, essential in the pathogenesis of UC. The release of various inflammatory factors was observed in the DSS-induced UC mouse model. The levels of IL-1β, IL-17, TNF-α, TGF-β1, INF-γ and IL-10 in colon tissues were assessed by ELISA. As shown in Figure 8, compared with normal mice, the levels of inflammatory factors IL-1β, IL-17, TNF-α, and INF-γ in DSS-induced ulcerative colitis mice were significantly increased. In contrast, anti-inflammatory factor IL-10 expression was significantly reduced. The proinflammatory cytokines in the API, API-Mn(II) complex, API-Mn(II)@HA nanoparticle and 5-ASA groups were downregulated. In this respect, IL-1β, IL-17, TNF-α, and INF-γ, were significantly reduced in the experimental groups, and the most significant reduction was found in the API-Mn(II)@HA BP group. On the contrary, the anti-inflammatory factor IL-10 was significantly upregulated. Interestingly, we found that the regulatory effect of API-Mn(II)@HA nanoparticles on cytokines was positively correlated with the dosage, further substantiating the therapeutic effect of API-Mn(II)@HA nanoparticles against inflammation.

### 3.9 Effects of API-Mn(II) complex and API-Mn(II)@HA nanoparticles on the colonic epithelial barrier

**FIGURE 9:**
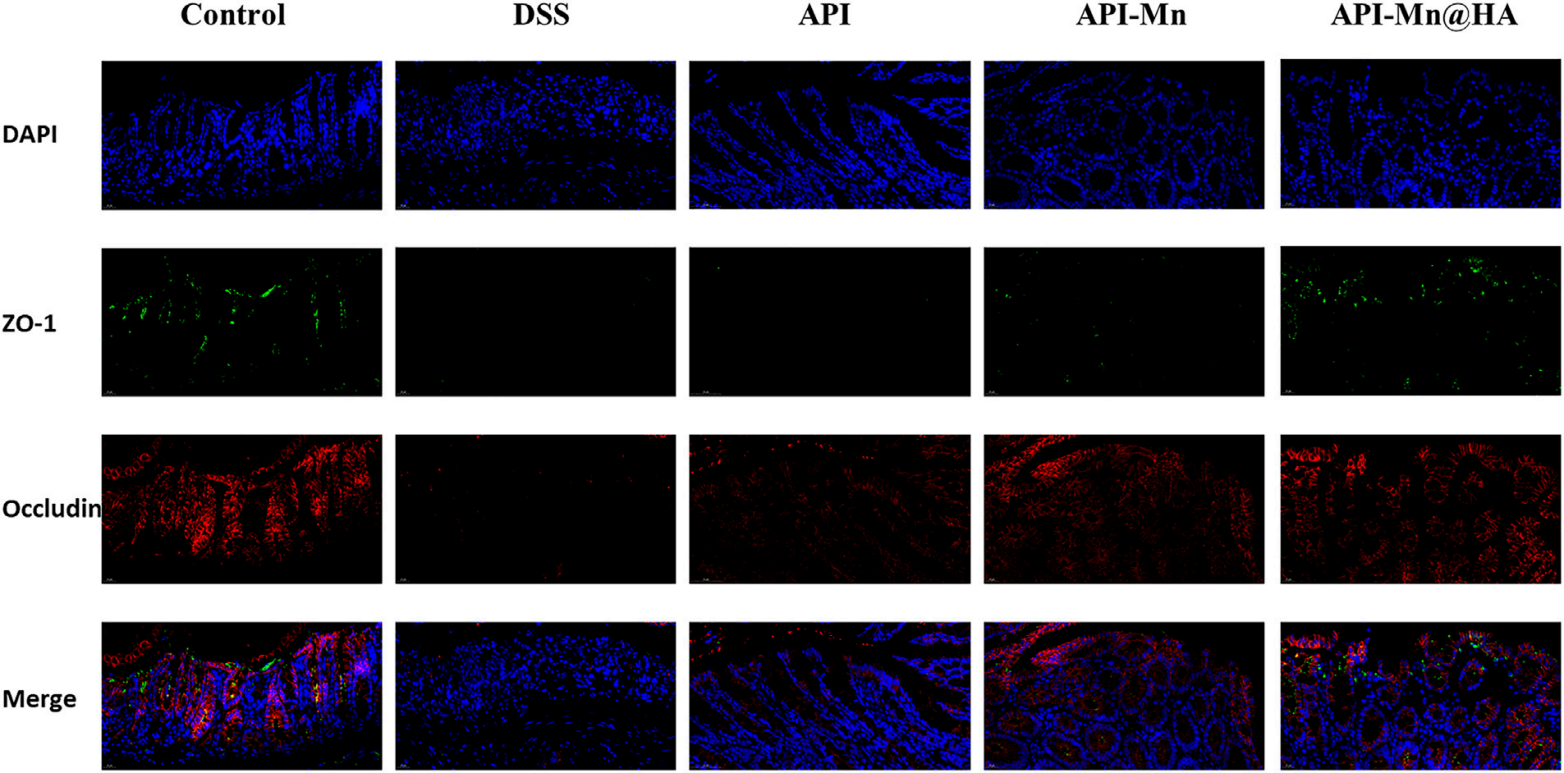
Immunofluorescence staining image of mice. Sections of tissue were stained with DAPI (blue) and ZO-1 (green) and Occludin (red).

ZO-1 and Occludin-1 are tight junction-associated proteins that play a key role in intestinal homeostasis. In Figure 9, occludin-1 (stained in red under fluorescence microscopy) was positively expressed in the control group, distributed in the apical membrane of intestinal epithelial cells, glandular epithelial cells and the cytoplasmic region on the proximal side of the membrane. Moreover, ZO-1 (stained in green) was positively expressed in the control group and distributed in the cytoplasm of intestinal epithelial and glandular epithelial cells near the medial membrane. Compared with the control group, the expression of occludin-1 and ZO-1 decreased in the DSS, API, API-Mn(II) complex and API-Mn(II)@HA nanoparticles groups. Moreover, the expression of occludin-1 and ZO-1 in the API-Mn(II)@HA NP group was higher than in the DSS group**.** In conclusion, we found that API-Mn(II)@HA nanoparticles could alleviate injury to tight junctions, increase their density and narrow the intercellular space, suggesting that API-Mn(II)@HA nanoparticles could repair cell-cell junctions between intestinal epithelial cells, especially tight junctions, damaged by DSS.

## 4 Conclusion

In this study, a coordination compound based on API and Mn^2+^ coated with HA was successfully prepared. The final product exhibited excellent water solubility and bioavailability. The molecular structure of the product was analyzed by infrared spectroscopy. Mice models were established to assess the efficacy of API in alleviating ulcerative colitis and its biosafety. We provided compelling evidence that the solubility and targeting were enhanced, accounting for the better therapeutic effect with API. Interestingly, the API-Mn(II)@HA nanoparticles exhibited the best therapeutic effect, better than the reference drug 5-ASA, suggesting that API-Mn(II)@HA nanoparticles yielded a stronger anti-inflammatory effect than 5-ASA, providing the foothold for the potential application of API in the clinical treatment of ulcerative colitis.

## Data Availability

The original contributions presented in the study are included in the article/supplementary material, further inquiries can be directed to the corresponding authors.

## References

[B1] Balmus I. M. Ciobica A. Trifan A. Stanciu C. (2016). The implications of oxidative stress and antioxidant therapies in Inflammatory Bowel Disease: Clinical aspects and animal models. Saudi J. Gastroenterol. 22 (1), 3. 10.4103/1319-3767.173753 26831601 PMC4763525

[B2] Collnot E-M. Ali H. Lehr C-M. (2012). Nano- and microparticulate drug carriers for targeting of the inflamed intestinal mucosa. J. Control. Release 161 (2), 235–246. 10.1016/j.jconrel.2012.01.028 22306429

[B3] Dai C. Zheng C. Q. Meng F. J. Zhou Z. Sang L. X. Jiang M. (2013). VSL#3 probiotics exerts the anti-inflammatory activity via PI3k/Akt and NF-κB pathway in rat model of DSS-induced colitis. Mol. Cell. Biochem. 374 (1-2), 1–11. 10.1007/s11010-012-1488-3 23271629

[B4] Funakoshi-Tago M. Nakamura K. Tago K. Mashino T. Kasahara T. (2011). Anti-inflammatory activity of structurally related flavonoids, apigenin, luteolin and fisetin. Int. Immunopharmacol. 11 (9), 1150–1159. 10.1016/j.intimp.2011.03.012 21443976

[B5] Ganjare A. Nirmal S. A. Patil A. N. (2011). Use of apigenin from *Cordia dichotoma* in the treatment of colitis. Fitoterapia 82 (7), 1052–1056. 10.1016/j.fitote.2011.06.008 21745550

[B6] Gao C. Liu L. Zhou Y. Bian Z. Wang Y. (2019). Novel drug delivery systems of Chinese medicine for the treatment of inflammatory bowel disease. Chin. Med. 14 (1), 23. 10.1186/s13020-019-0245-x 31236131 PMC6580650

[B7] Huang Y. Liu Z. Liu C. Ju E. Zhang Y. Ren J. (2016). Self-assembly of multi-nanozymes to mimic an intracellular antioxidant defense system. Angew. Chem. Int. Ed. Engl. 55 (23), 6758–6762. 10.1002/ange.201600868 27098681

[B8] Lamprecht A. Yamamoto H. Takeuchi H. Kawashima Y. (2005). Nanoparticles enhance therapeutic efficiency by selectively increased local drug dose in experimental colitis in rats. J. Pharmacol. Exp. Ther. 315 (1), 196–202. 10.1124/jpet.105.088146 15980057

[B9] Li H. X. Wen L. J. Chen L. Z. Zhai R. R. Cai L. J. (2016). Synthesis of schiff base metal derivatives of apigenin and their antioxidant activity. Mod. Food Sci. Technol. 32 (7), 164–9294. 10.13982/j.mfst.1673-9078.2016.7.026

[B10] Linglong M. A. Xiaoshuang L. I. Zhongkun L. U. Zhu L. Chen S. Yinlian F. U. (2019). Synthesis and free radical scavenging activities of apigenin copper(Ⅱ) complex. Food Sci. Technol. 44 (06), 275–281. 10.13684/j.cnki.spkj.2019.06.046

[B11] Löwenberg M. D’Haens G. (2013). Novel targets for inflammatory bowel disease therapeutics. Curr. Gastroenterol. Rep. 15 (2), 311. 10.1007/s11894-012-0311-3 23314806

[B12] Márquez-Flores Y. Villegas I. Cárdeno A. Rosillo Má Alarcón-de-la-Lastra C. (2016). Apigenin supplementation protects the development of dextran sulfate sodium-induced murine experimental colitis by inhibiting canonical and non-canonical inflammasome signaling pathways. J. Nutr. Biochem. 30, 143–152. 10.1016/j.jnutbio.2015.12.002 27012631

[B13] Molodecky N. A. Soon I. S. Rabi D. M. Ghali W. A. Ferris M. Chernoff G. (2012). Increasing incidence and prevalence of the inflammatory bowel diseases with time, based on systematic review. Gastroenterology 142 (1), 46–54. 10.1053/j.gastro.2011.10.001 22001864

[B14] Pan H. Wang F. Zhuang R. Zhang J. Fang H. Jianjun X. I. (2014). Study on preparation of apigenin-magnesium complex. Chin. Archives Traditional Chin. Med. 10.13193/j.issn.1673-7717.2014.02.039

[B15] Pan X. Shao Y. Wang F. Cai Z. Liu S. Xi J. (2020). Protective effect of apigenin magnesium complex on H2O2-induced oxidative stress and inflammatory responses in rat hepatic stellate cells. Pharm. Biol. 58 (1), 553–560. 10.1080/13880209.2020.1772840 32544362 PMC8641681

[B16] Sakai S. Nishida A. Ohno M. Inatomi O. Andoh A. Sugimoto M. (2019). Astaxanthin, a xanthophyll carotenoid, prevents development of dextran sulphate sodiuminduced murine colitis. J. Clin. Biochem. Nutr. 64 (1), 66–72. 10.3164/jcbn.1847 30705514 PMC6348411

[B17] Shen R. Liu M. Zhu X. D. Tan W. J. Wang Y. (2018). Experimental research progress on traditional Chinese medicine treatments for ulcerative colitis. Chin. Traditional Herb. Drugs 49 (7), 1721–1725. 10.7501/j.issn.0253-2670.2018.07.035

[B18] Yao J. Cheng Y. Zhou M. Zhao S. Lin S. Wang X. (2019). ROS scavenging Mn3O4 nanozymes for *in vivo* anti-inflammation. Chem. Sci. 9, 2927–2933. 10.1039/c7sc05476a PMC5915792 29732076

[B19] Yu Z. Zhanzhan Z. Zheng P. Yang L. (2021). Advanced bioactive nanomaterials for biomedical applications. Exploration 1 (3), 20210089. 10.1002/EXP.20210089 PMC10191050 37323697

[B20] Zhao H. Cheng N. Zhou W. Chen S. Cao W. Gao H. (2019). Honey polyphenols ameliorate DSS-induced ulcerative colitis via modulating gut microbiota in rats. Mol. Nutr. Food Res. 63 (23), 1900638. 10.1002/mnfr.201900638 31533201

[B21] Zheng C. Li M. Ding J. (2021). Challenges and opportunities of nanomedicines in clinical translation. BIO Integr. 2, 57–60. 10.15212/bioi-2021-0016

[B22] Zhu Z. Wang Y. L. Yang Y. P. Shi-Zhuo X. U. Dou J. J. Guo B. N. (2012). Synthesis of new hyaluronic metal complexes and investigation of biological activity. Trans. Beijing Inst. Technol. 32 (11), 1200–1204. 10.15918/j.tbit1001-0645.2012.11.002

