# Supplementary material for: Apigenin-Mn(II) loaded hyaluronic acid nanoparticles for ulcerative colitis therapy in mice
Source: Front Chem. 2022 Jul 22;10:969962. doi: 10.3389/fchem.2022.969962 (PMC9354835; doi:10.3389/fchem.2022.969962)
Supplement: Supplementary file 1 [file DataSheet1.docx]

Table S1 Criteria for scoring the DAI

| Weight loss (% of initial body weight) | Stool consistency | Bleeding | Score |
| --- | --- | --- | --- |
| ＜1 | Normal pellets | Normal | 0 |
| 1～5 | Slightly loose feces | — | 1 |
| 5～10 | Loose feces | Bloody | 2 |
| 10～20 | lightly loose feces | — | 3 |
| ＞20 | Watery diarrhea | Blood in whole colon | 4 |

Table S2 Histological colitis score

| Score | Inflammation | | Extent | | | Crypt Damage | |
| --- | --- | --- | --- | --- | --- | --- | --- |
| 0 | None | | None | | | None | |
| 1 | Slight | | Mucosa | | | Basal 1/3 damaged | |
| 2 | Moderate | | Mucosa and submucosa | | | Basal 2/3 damaged | |
| 3 | Severe | | Transmural | | | Only surface epithelium intact | |
| 4 |  | |  | | | Entire crypt and epithelium lost | |
| Area involved (%) | 0 | 1–25 | | 26–50 | 51–75 | | 76–100 |
| Factor | 0 | 1 | | 2 | 3 | | 4 |

Table S3 Factors and levels of Box —Behnken tests

|  | A | B | C |
| --- | --- | --- | --- |
| Level | pH | Time/h | Temperature/ ^o^C |
| -1 | 7 | 3 | 50 |
| 0 | 8 | 4 | 60 |
| 1 | 9 | 5 | 70 |

Table S4 Results of Box-Behnken tests

| No. | Response value | | | |
| --- | --- | --- | --- | --- |
|  | pH | Time/h | Temperature/^o^C | Yield |
| 1 | 7.00 | 5.00 | 60.00 | 23.25 |
| 2 | 8.00 | 4.00 | 60.00 | 81.35 |
| 3 | 8.00 | 4.00 | 60.00 | 80.37 |
| 4 | 8.00 | 5.00 | 50.00 | 54.15 |
| 5 | 7.00 | 4.00 | 50.00 | 16.61 |
| 6 | 7.00 | 4.00 | 70.00 | 22.10 |
| 7 | 9.00 | 4.00 | 50.00 | 23.54 |
| 8 | 7.00 | 3.00 | 60.00 | 21.88 |
| 9 | 8.00 | 3.00 | 50.00 | 53.15 |
| 10 | 8.00 | 4.00 | 60.00 | 84.68 |
| 11 | 9.00 | 3.00 | 60.00 | 32.83 |
| 12 | 9.00 | 4.00 | 70.00 | 37.78 |
| 13 | 9.00 | 5.00 | 60.00 | 32.85 |
| 14 | 8.00 | 3.00 | 70.00 | 66.10 |
| 15 | 8.00 | 4.00 | 60.00 | 80.37 |
| 16 | 8.00 | 4.00 | 60.00 | 85.00 |
| 17 | 8.00 | 5.00 | 70.00 | 65.10 |

Table S5 Variance analysis of response surface experiments results

| Source | Sum of  Squares | | df | Mean Square | F Value | p-value | statistics |
| --- | --- | --- | --- | --- | --- | --- | --- |
| Model | 10576.06 | 9 | | 1175.12 | 339.38 | < 0.0001 | **** |
| A-PH | 232.85 | 1 | | 232.85 | 67.25 | < 0.0001 | **** |
| B-Time | 0.24 | 1 | | 0.24 | 0.070 | 0.7993 |  |
| C-Temperature | 237.95 | 1 | | 237.95 | 68.72 | < 0.0001 | **** |
| AB | 0.46 | 1 | | 0.46 | 0.13 | 0.7275 |  |
| AC | 19.14 | 1 | | 19.14 | 5.53 | 0.0510 |  |
| BC | 1.00 | 1 | | 1.00 | 0.29 | 0.6076 |  |
| A^2^ | 8388.3 | 1 | | 8388.37 | 2422.62 | < 0.0001 | **** |
| B^2^ | 422.49 | 1 | | 422.49 | 122.02 | < 0.0001 | **** |
| C^2^ | 680.40 | 1 | | 680.40 | 196.50 | < 0.0001 | **** |
| Residual | 24.24 | 7 | | 3.46 | 0.18 | 0.9018 |  |
| Lack of Fit | 2.95 | 3 | | 0.98 |  |  |  |
| Pure Error | 21.29 | 4 | | 5.32 |  |  |  |
| Cor Total | 10600.29 | 16 | |  |  |  |  |
|  |  |  | | R^2^_Adj_=0.9948 | | S/N=46.251 | |

Tab S6 Yield of complex with different mix ratio and amount of hyaluronic acid

| Mix proportion | Dosage of complex | Dosage of HA | Product quality | Yield |
| --- | --- | --- | --- | --- |
| 1:3 | 5mg | 17.1mg | 17.7mg | 80.2% |
| 1:6 | 5mg | 34.2mg | 35.2mg | 89.7% |
| 1:9 | 5mg | 51.3mg | 43.7mg | 77.6% |


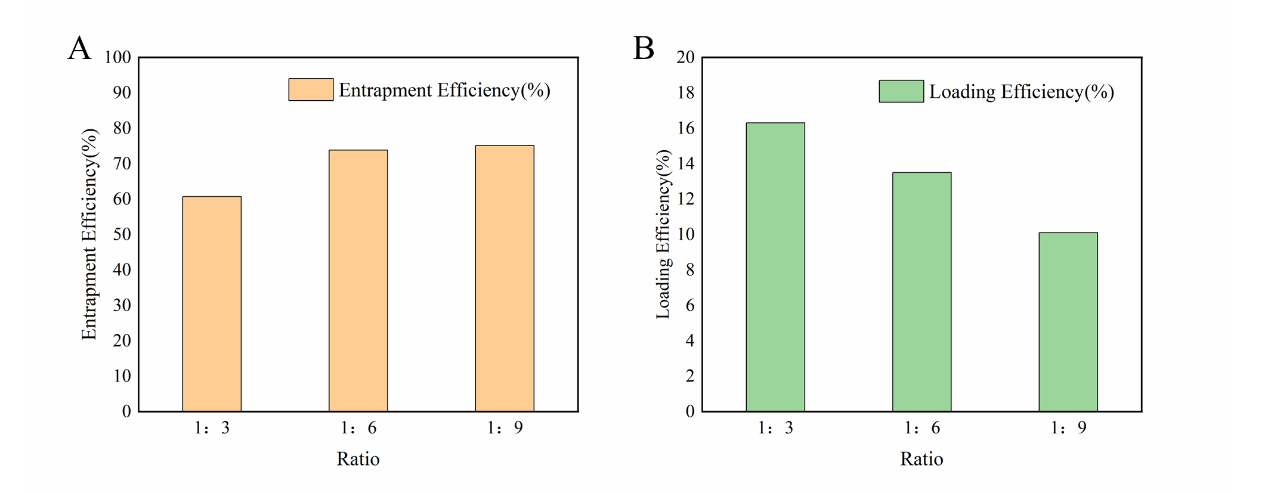


Fig S1 The encapsulation rate (A) and drug loading rate (B) of drug with different HA ratio.


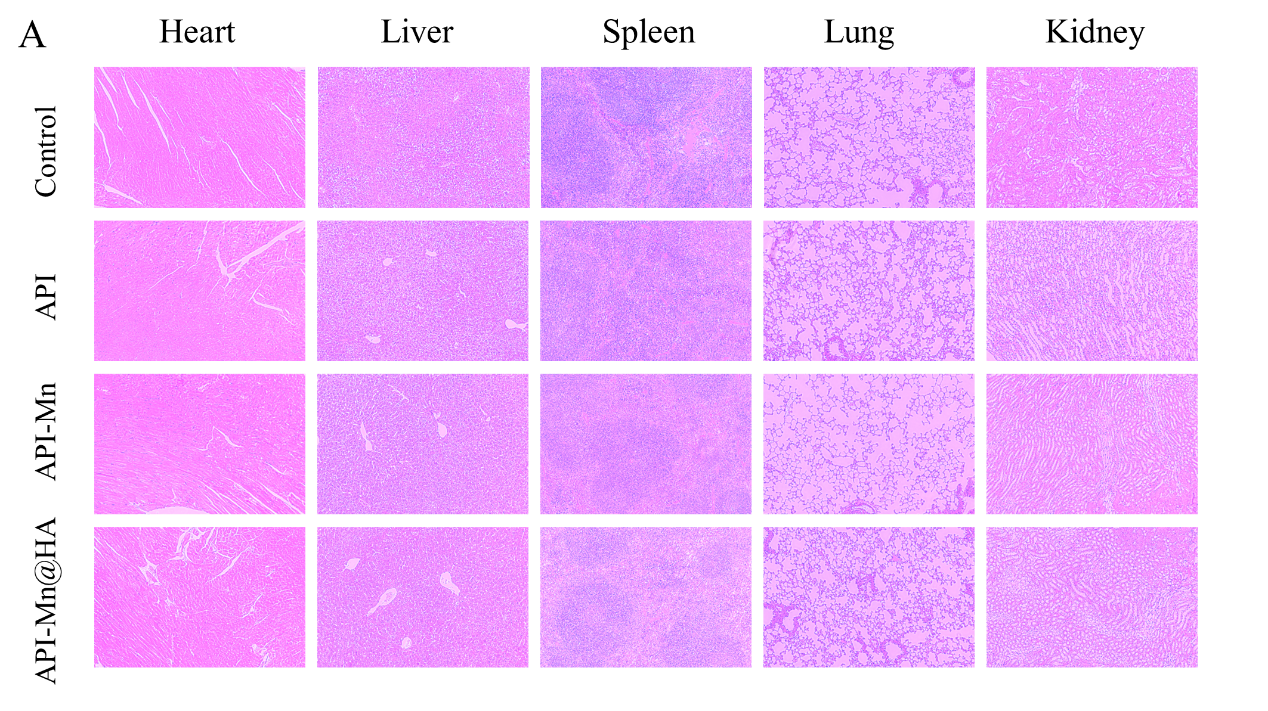

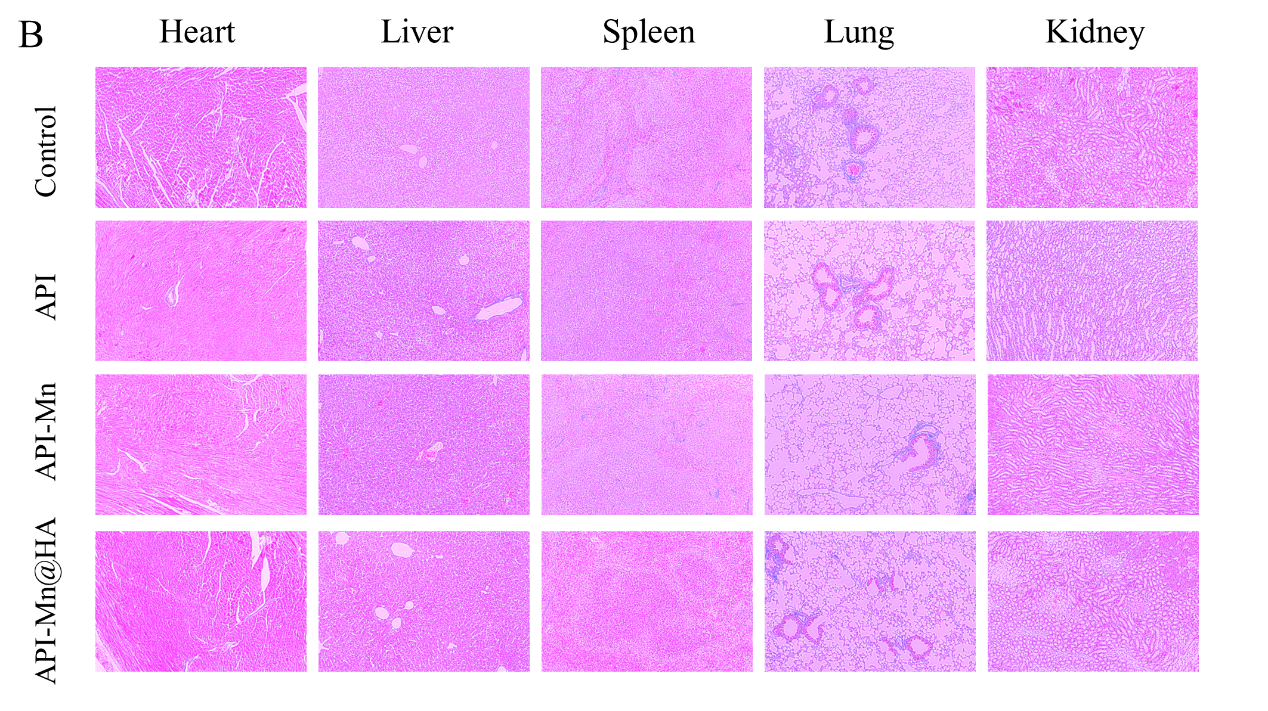


Fig S2 HE (A) and Masson (B) staining of mouse major organs


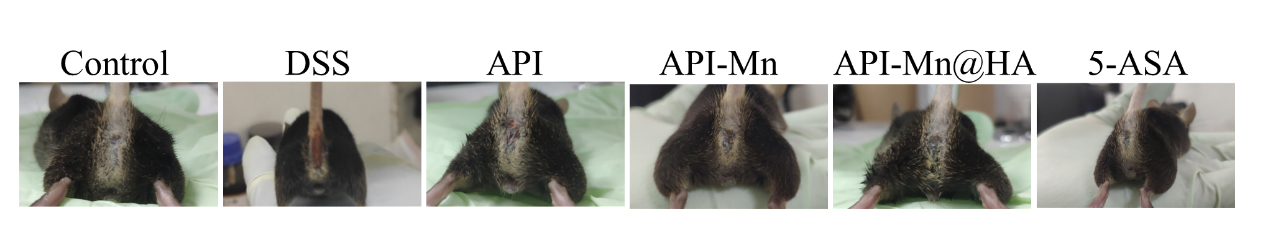


Fig S3 Pictures of mice with hematochezia


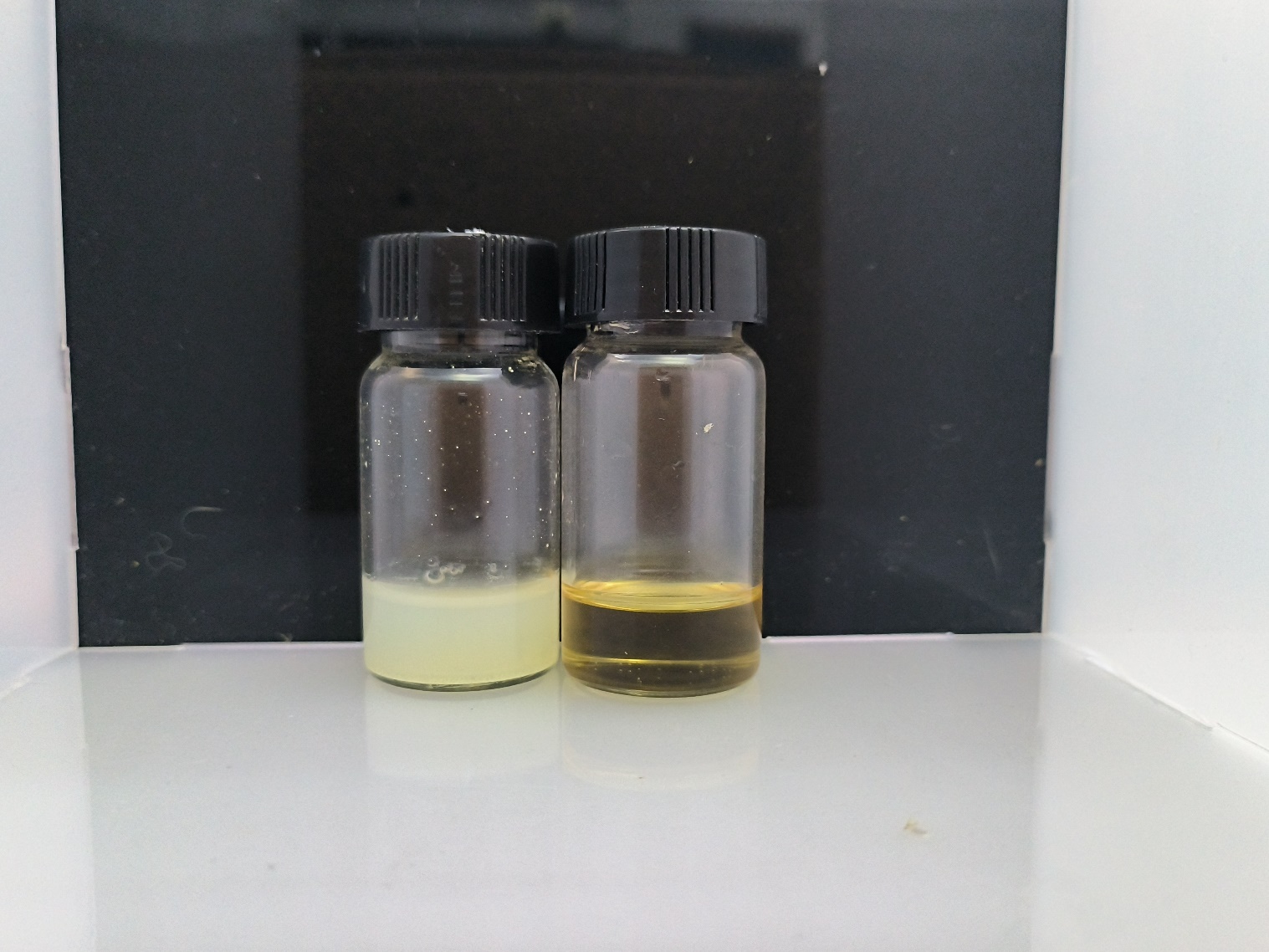


Fig S4 Dispersion of API-Mn@HA (Right) and API (Left) in water.
